# Hands Down: Cognate Effects Persist During Written Word Production

**DOI:** 10.3389/fpsyg.2021.647362

**Published:** 2021-07-05

**Authors:** Evy Woumans, Robin Clauws, Wouter Duyck

**Affiliations:** ^1^Department of Translation, Interpreting and Communication, Ghent University, Ghent, Belgium; ^2^Department of Experimental Psychology, Ghent University, Ghent, Belgium

**Keywords:** cognate effect, typewriting, picture naming, sentence context, word production, language non-selective activation, bilingualism

## Abstract

Words that share form and meaning across two or more languages (i.e., cognates) are generally processed faster than control words (non-cognates) by bilinguals speaking these languages. This so-called cognate effect is considered to be a demonstration of language non-selectivity during bilingual lexical access. Still, research up till now has focused mainly on visual and auditory comprehension. For production, research is almost exclusively limited to speech, leaving written production out of the equation. Hence, the goal of the current study was to examine whether bilinguals activate representations from both languages during typewriting. Dutch-English bilinguals completed second-language written sentences with names of displayed pictures. Low-constraint sentences yielded a cognate facilitation effect, whereas high-constraint sentences did not. These findings suggest that co-activation of similar words across languages also occurs during written production, just as in reading and speaking. Also, the interaction effect with sentence constraint shows that grammatical and semantic sentence restrictions may overrule interlingual facilitation effects.

## Introduction

If a bilingual reads, hears, or produces a word, do they activate a representation of that word in one or multiple languages? Much research has sought to answer this question and different theories have been put to the test. At present, there is general consensus on an integrated lexicon (see Brysbaert and Duyck, 2010) or a segregated lexicon that is activated in parallel (Dijkstra et al., 2019). Evidence to support this idea comes from studies employing interlingual homonyms or cognates to demonstrate language non-selective activation (e.g., Dijkstra et al., 1999; Costa et al., 2000; Lagrou et al., 2011a, b). Interlingual homonyms are words that share spelling but not meaning within or across languages, such as *boot* in English (meaning footwear) and *boot* in Dutch (meaning a vessel). We may distinguish between homographs (words that are typographically similar) and homophones (words that are phonologically similar). In contrast, cognates are words that share form (completely or predominantly) and meaning across languages, such as the Dutch and English word *film*. The cognate facilitation effect states that cognates are read (Cop et al., 2017), heard (Blumenfeld and Marian, 2007), and spoken (Costa et al., 2000) faster than non-cognates by bilinguals speaking those specific languages, whereas cross-linguistic homophones seem to interfere with bilingual language processing, in listening (Lagrou et al., 2015) and reading (Dijkstra et al., 1999), just like homographs (see Dijkstra et al., 2000, for reading; Lagrou et al., 2011a, b; for listening; Jared and Szucs, 2002, for speaking).

Findings such as these show that words of each language can be activated in both first (L1) and second (L2) language processing and support both an integrated lexicon and language non-selective access. Still, looking at the body of established research on bilingual lexical access, it is clear that lot of research has mainly focused on word recognition (Caramazza and Brones, 1979; Dijkstra et al., 1999; Spivey and Marian, 1999; Schwartz et al., 2007; Lagrou et al., 2011a), and to a lesser extent on word production (Costa et al., 2000; Hoshino and Kroll, 2008). Studies on cross-lingual activation during written word production, however, are virtually non-existent. Hence, it was the current study’s aim to fill in the gap for written word production. In addition, studies on word recognition and speaking mostly investigated isolated word processing, although the grammatical and semantic restrictions that arise from sentences may be important modulators of cross-lingual lexical activation. It is only later that sentence studies were carried out in these modalities (e.g., Duyck et al., 2007; Van Assche et al., 2009). Here, as a second aim, we will also investigate the impact of sentence constraint for written word production, and more specifically for typewriting.

### Examining Lexical Access Using Isolated Word Paradigms

Research into bilingual lexical access has often employed lexical decision paradigms to obtain more insight into bilingual language processing. A pioneering study by Caramazza and Brones (1979) had Spanish-English subjects classify strings of letters as either English or Spanish words or non-words. Also included were cognates, both in the blocked (L1 or L2 only) and mixed (L1 and L2) conditions. A cognate facilitation effect was found in the L2 blocked condition and in the mixed condition. Since then, this facilitation effect has been replicated by a number of studies (e.g., Dijkstra et al., 1999; Schwartz et al., 2007). Most notably, facilitatory effects of cross-linguistic overlap also appear to be progressive. In a study among trilingual subjects, Lemhöfer et al. (2004) employed both words that were cognates across two languages as well as words that were cognates across three languages. When performing a lexical decision task in their third language German, the Dutch-English-German trilinguals responded faster for so-called “double cognates” (with overlap in Dutch and German) as opposed to control words, but even faster responses were produced for triple cognates (with overlap in Dutch, German, and English). These findings again supported the view of language non-selective access, and furthermore implied that all languages known to an individual may affect word recognition and activation.

In a more elaborate study not only constricted to cognates but also containing homonyms, Dijkstra et al. (1999) explored lexical decision performance on English words that varied according to the degree with which they shared orthography, phonology, semantics, or some combination of the three codes with Dutch words. Dutch-English bilinguals showed faster response latencies for words that shared orthography (i.e., homographs) or a combination of orthography and semantics (i.e., cognates), supporting the language non-selective access hypothesis. In contrast, recognition latencies were delayed when words shared only phonology (i.e., homophones), which the authors explained as an inhibitory effect. A given letter string may activate all compatible phonological codes independent of language (e.g., Brysbaert et al., 1999), but the activated non-identical phonological lexical representations may compete at a lexical level, resulting in a delayed identification of the item in the target language (i.e., lateral inhibition).

Important to note, however, is that interlingual homographs and even cognates may also serve as inhibitors of lexical access when presented under constricting circumstances. When Dutch-English bilinguals were asked to respond to interlingual homographs in an English lexical decision task with only English words in the stimulus list, latencies for homographs and control words did not differ. However, when half of the non-words were replaced by Dutch words requiring a no-response in this task, latencies for homographs slowed down substantially (Dijkstra et al., 1998). Similarly, Dutch-English bilinguals demonstrated a cognate inhibition effect when performing an English lexical decision task where the non-words were replaced by Dutch words (Vanlangendonck et al., 2020). This adaptation in context (purely English versus English mixed with Dutch) completely reversed the cognate facilitation effect. In contrast, cognate facilitation does seem to uphold in mixed conditions where stimuli in both languages require a yes-response (i.e., generalized lexical decision) (Lemhöfer and Dijkstra, 2004).

Although the abovementioned studies were restricted to visual word recognition, evidence suggests that also for spoken word recognition, lexical access is non-selective. For instance, a study by Lagrou et al. (2011a) provided confirmation for the findings of Dijkstra et al. (1999), when they demonstrated a similar delay in response latencies when bilinguals had to respond to homophones in an auditory lexical decision task. Furthermore, an early study by Spivey and Marian (1999) hypothesized that bilinguals might be distracted by words from their one language when doing a task in their other language, if the initial phonemes of the words overlap. This hypothesis was based on previous findings from monolingual research, which suggest that when subjects produce a spoken word, all words starting with the same sounds are initially activated (e.g., Marslen-Wilson, 1987). Employing a visual world paradigm, Spivey and Marian presented Russian-English bilinguals with verbal instructions, which told them to pick up a stamp (e.g., *marku* in Russian) while one of the distractor objects was a *marker* (a word starting with the same phonemes). Confirming the author’s hypothesis, the subjects looked more toward the marker than any of the other two objects. Similar results were found when English was the language of instruction.

Although a study by Weber and Cutler (2004) was able to replicate the cross-language finding of Spivey and Marian (1999) when instructions were given in L2, this was not the case for L1. The authors explained the difference by stating that Spivey and Marian had tested their participants in an L2 environment (Russian students studying at an American university), whereas Weber and Cutler studied their participants in an L1 environment. However, Lagrou et al. (2013) rightly noted that the phoneme overlap between the targets and the distractors in the study by Weber and Cutler was very small (i.e., often only one phoneme). When these authors repeated the experiment with stimuli that elicited more cross-lingual activation (i.e., words with more overlap), they were able to confirm cross-language activation for spoken word recognition also in an L1 context.

Whereas the studies described above focused on word recognition, there is also evidence that confirms non-selective access in word production. Costa et al. (2000), for instance, found that Catalan-Spanish bilinguals were faster at naming cognate words in a picture naming task, which they performed in their L2 Spanish. Although Catalan and Spanish share many linguistic features, the finding has also been replicated among different-script bilinguals. In a study by Hoshino and Kroll (2008), Japanese–English bilinguals named cognate and non-cognate pictures in their L2 English and they also demonstrated a cognate facilitation effect. The authors concluded that this outcome implies there is cross-language activation of phonology even for different-script bilinguals.

### Does Sentence Constraint Alter Bilingual Lexical Access?

Until now, we have primarily focused on studies reporting cognate and interlingual homograph effects on words presented in isolation, as this was the prime interest of literature. However, the outcome of these studies may not be representative for natural language processing, as language context (e.g., through text and situation) usually provides bilinguals with a clear cue for which language requires activation, or even which word. Looking at sentence context, one may argue that if an unfolding sentence is predictive of an upcoming word, cognate effects for this particular word may disappear. Indeed, previous work shows that when sentences are low semantically constraining and therefore not predictive, cognate effects still appear, whereas this is not the case for highly constraining and hence predictive sentences (e.g., Schwartz and Kroll, 2006; Van Hell and De Groot, 2008; Libben and Titone, 2009). These findings led to the conclusion that language non-selectivity may be restricted by semantically constraining contexts. However, due to the overwhelming body of research supporting language non-selective access (see Brysbaert and Duyck, 2010), a more plausible explanation is that highly predictive target words, cognate and otherwise, will already be activated before a lexical decision needs to be made, thereby diminishing the cognate facilitation effect.

Employing a slightly different paradigm with picture naming instead of lexical decision within sentence context, Starreveld et al. (2014) found that naming latencies were sped up by the sentence context, but cognate effects still remained, especially when naming occurred in L2. In L1, however, the cognate effect was only present in non-predictive sentences. In addition, processing of interlingual homophones also seems unaltered by semantic constraint, even in L1. Lagrou et al. (2013) had Dutch-English bilingual participants perform lexical decision on the last word of a sentence. When this word was an interlingual homophone (e.g., /li:f/: *lief* - sweet - in Dutch vs. *leaf* in English), response latencies were slowed down in both language conditions. Although still present, the effect did, however, reduce in size when the homophones were presented in highly semantically constraining sentences. The authors thus concluded that sentence constraint may influence word recognition, but it does not necessarily eliminate cross-lingual lexical interactions.

The story becomes even more interesting when considering studies that have looked at a more natural way of language processing. For instance, Van Assche et al. (2011) recorded Dutch-English bilinguals’ eye movements while they read cognates and control words embedded in low and high semantically constraining sentences presented in their second language. Both early and late eye-movement measures yielded cognate facilitation, for low as well as highly constraint sentences. The authors viewed these results as evidence in support of a limited role for top-down influences of semantic constraints on lexical access in both early and later stages of bilingual word recognition. And, more recently, cognate facilitation was even obtained in bilinguals reading an entire book, both for L1 and L2 reading (Cop et al., 2017).

The discrepancies between this and other studies may of course find their origin in the paradigms that are being used. Indeed, when subjects are asked to perform a lexical decision task, response latencies are influenced not only by the time it takes to recognize a word, but also by a decision-making component and even the motor processes required to deliver the manual response (Pinet et al., 2016). Also response strategies that favor either accuracy or speed may play a role. Studies that have compared lexical decision databases with natural reading corpora indeed found that results diverged considerably across paradigms (Kuperman et al., 2013; Dirix et al., 2019). Even across eye tracking corpora correlations of reading times were low whereas within-task reliability was high, illustrating a strong effect of language context. Yet, when aggregating eye tracking measures across multiple representations and contexts, eye tracking measures increasingly converged with lexical decision data, indicating that task-specific language context has a crucial impact on word-level effect manifestation (see Dirix et al., 2019 for a more elaborate view).

### Modeling Bilingual Lexical Access

Theoretical accounts of cross-lingual activation are provided within bilingual language processing models such as the BIA model (Bilingual Interactive Activation model; Dijkstra and Van Heuven, 1998), and its successor BIA+ (Dijkstra and Van Heuven, 2002). These models of word recognition propose that the visual presentation of a word leads to co-activation of many word candidates from different languages that are similar to the input (i.e., orthographic neighbors). This describes the process of language non-selective lexical access. The orthographic representations will subsequently activate their semantic representations (i.e., meaning) (see, for instance, Grainger, 2008) and their phonological representations (e.g., Coltheart et al., 2001). Within this framework, the processing of cognates may be understood by assuming that both orthographic representations of a cognate become activated by the input and subsequently send converging activation to a shared semantic representation (e.g., Vanlangendonck et al., 2020), which leads to faster processing of the word.

However, in order to provide a general implemented account of word form and meaning retrieval during word production as well as recognition, a localist-connectionist model “Multilink” has recently been developed (Dijkstra et al., 2019). This model integrates the basic assumptions of BIA+ together with the basic architecture of the Revised Hierarchical Model (RHM; Kroll and Stewart, 1994; but see both Brysbaert and Duyck, 2010 and Dijkstra et al., 2019 for a clarification of this model ad its issues), and simulates recognition and production of cognates and non-cognates in tasks such as monolingual or bilingual lexical decision, word naming, and word translation production. Multilink is based on a number of assumptions, such as the supposition that the activation of competitors in L1 and L2 directly depends on the orthographic overlap between the input word and stored lexical representations, as operationalized by their Levenshtein distance (Levenshtein, 1966). It also assumes that word candidates compete only at a response choice level but not in terms of lateral inhibition; and that orthographic lexical representations only indirectly (via semantics) cross-linguistically linked. Running simulations on the retrieval of cognates, the model demonstrated that lexical activation spreading from orthographic representations to their (same-language) phonological representations might account for the cognate facilitation effect in word production. This is in line with earlier predictions made by, for instance, Strijkers et al. (2010), who suggested that the overlap between L1 and L2 phonology in cognates results in co-activation of both translation equivalents. This, in turn, culminates in a strong connection between both lexical representations, which is no longer mediated by phonological access. We must, however, note that the current models only simulate single word production and not words within context.

### The Current Study

It is striking that a plethora of research exists on bilingual reading, listening, and speaking, whereas virtually no study has assessed written word production. This mimics the monolingual literature that is also much scarcer in terms of writing, largely because fewer good paradigms exist to assess timing of subprocesses. Still, the same questions pose themselves, such as those on cross-lingual activation. Although spoken and written word production are very distinct activities (i.e., the former requires the utterance of phonemes through vocalization and mouth control, whereas the latter requires the formation of graphemes through control of the hands), lexical access should nevertheless be similar up to a certain stage. Indeed, a basic assumption is that conceptual and lexical processes are shared between spoken and written modalities, whereas post-lexical processes (i.e., phonological/orthographic selection and motor control) are different (e.g., Hillis et al., 1990; Perret and Laganaro, 2013). This implies that also during writing, bilinguals should benefit from a cognate facilitation effect. Taking into account the findings of Starreveld et al. (2014) for spoken production, we might assume that cognate facilitation in L2 written production should even remain present in highly constraining conditions. However, to the best of our knowledge, this assumption has never been tested. The present study thus set out to examine this issue.

In addition, we wanted to assess the impact of sentence context on such cross-lingual activation. Employing a similar methodology to that of Starreveld et al. (2014), we presented our Dutch-English participants with targets embedded within either low or high constraint sentences formulated in their L2. The targets were cognates and non-cognates presented as pictures, which participants had to name by typing in their responses. If the process of written word production is similar to spoken word production and written/spoken word recognition, a clear cognate facilitation effect should occur, at least in the low constraint context.

## Materials Ans Methods

### Participants

Twenty students in their first Bachelor in Psychology, aged between 18 and 24 (*M* = 18.8 years; 16 females) participated in the study in exchange for course credit. Prerequisites for participation entailed having Dutch as the native language and a score of at least 70% on the English version of LexTALE (Lemhöfer and Broersma, 2011). Participants were also required to have normal or corrected vision. All individuals signed an informed consent and had the option to exit the experiment at any time of their choosing.

### Materials

One hundred and fifty sentences were presented to participants in their L2 English. These were typewritten, but contained one image presenting one of 25 cognate words, one of 25 matched control words, or one of 25 filler words (see Supplementary Appendix A). All images depicting cognates and controls were obtained from the database of Severens et al. (2005). Out of the database containing 590 standardized black-and-white line drawings with picture naming norms in Dutch, we selected those images with a name agreement higher than 75%. Participants were therefore not exposed to the pictures prior to the experiment. Cognate and control words were matched on initial letter, word length (cf. Bates et al., 2003), and word frequency. We employed the SUBTL frequency norms from the SUBTLEXUS corpus (Brysbaert and New, 2009). This procedure provided us with 25 cognates and 25 control words. For a full list of all the words used in the experiment, see Supplementary Appendix A. With regard to the cognates, we employed both identical (*N* = 14) and non-identical (*N* = 11) words, with a mean Levenshtein Distance of 0.64 (*SD* = 0.81) based on orthographic overlap.

All sentences had a similar structure, with the target picture presented in the middle of the sentence (see Table 1 for an example). For a full list of all the sentences used, see Supplementary Appendix B. To obscure the purpose of our study, we also included 50 filler sentences. Each picture was presented two times; once in a highly predictive (high constraint) context and once in a non-predictive (low constraint) context. First key stroke latencies were measured (cf. Baus et al., 2013) as the onset of lexical access. There were five blocks of 30 sentences; each containing 10 sentences with a cognate word, 10 sentences with a control word, and 10 sentences with a filler word. All participants saw the 150 sentences in a balanced randomised order and no picture was presented twice in one block.

**TABLE 1:** Examples of each condition that will be analyzed.

Sentence type	Word type	Example Sentence
Low constraint	Control	The baron ordered his servant to bring him a PLATE so he could throw it at the wall.
	Cognate	Jeff is very proud of his PIANO because his grandfather made it for him.
High constraint	Control	His mum cooked dinner and put some potatoes and a pork chop on his PLATE before sitting down herself.
	Cognate	The white keys are larger than the black keys on a PIANO because they are used more often.

### Procedure

Participants were seated in front of a computer with their head at approximately 60 cm distance from the monitor. Participants employed a QWERTY keyboard and started the experiment with a short practice phase of 15 warm up trials, containing sentences from all conditions. After the practice phase, the experiment started with instructions presented in the middle of the screen. In addition, verbal instruction was provided. Participants were told to rest their hands in front of the keyboard when they did not have to type. In order to ensure they paid attention to the sentences and not just to the pictures, we clarified that occasionally they would be asked the question: “What was stated in the previous sentence?.” This question appeared on the screen 5 times per block. Participants had to type in their response and press enter to continue the experiment.

After the instructions were read, a fixation cross (+) appeared for 500 ms in the middle of the screen, after which the first word appeared. Participants controlled the speed of reading themselves by pressing the space bar. When the picture was presented on the screen, participants were able to type the name of the depicted object and saw their own text appear directly underneath the picture. They were allowed to correct themselves, as long as they were still on the picture and had not pressed enter. Pressing the enter key led them to the rest of the sentence. After each trial, an empty screen was presented for 1000 ms before the next trial started.

At the end of the experiment, participants were thanked and awarded with course credit. On average, the experiment took about 50–60 min to complete.

## Results

There were 500 observations per condition across participants. No participant answered more than 5 comprehension questions incorrectly, so no participants were excluded from the analysis. The data were trimmed removing incorrect responses (e.g., faulty first strokes) and response times longer than 2.5 standard deviations from the average response time. This procedure eliminated 2.26% of the data, resulting in 350 observations per condition. Incorrect responses in the form of faulty first strokes were common, as the standard use of keyboard in Belgium is not QWERTY but AZERTY. However, since we removed all matched trials as well (i.e., the word with faulty stroke and its matched cognate/control in both high and low constraint condition), this should not influence our results.

We employed a 2 × 2 factorial design in our experiment. Our variables and their dimensions were Sentence Constraint (Low vs. High) and Word Type (Control vs. Cognate). A two-way repeated measures analysis of variance (ANOVA) was conducted on the means per condition to examine the main effects of Sentence Constraint and the Word Type, and their interaction effect on the first stroke latency of typing a word. We employed a multivariate approach, using Wilks’ Lambda. There was a significant main effect of Sentence Constraint [*F*_1_(1,19) = 18.31, *p* < 0.001; *F*_2_(1,48) = 5.86, *p* = 0.019], with faster responses in high constraint sentences. The main effect of Word Type did not reach significance [*F*_1_(1,19) = 3.70, *p* = 0.060; *F*_2_(1,48) = 0.10, *p* = 0.758]. There was, however, a significant interaction between Sentence Constraint and Word Type [*F*_1_(1,19) = 35.09, *p* < 0.001; *F*_2_(1,48) = 9.92, *p* = 0.003], with shorter response latencies to cognates in low constraint sentences and to control words in high constraint sentences.

Further analyses using the results from the multivariate approach revealed that the level of sentence constraint has a significant effect on first stroke latency, but only for control words [*F*_1_(1,38) = 22.159, *p* < 0.001; *F*_2_(1,23) = 3.80, *p* = 0.001], with shorter latencies in the high constraint condition. In addition, there was a significant effect of Word Type on first stroke latencies, but only in low constraint sentences [*F*_1_(1,38) = 12.463, *p* = 0.010; *F*_2_(1,24) = 2.55, *p* = 0.018], with shorter response latencies to cognates. Six paired *t*-tests were conducted with the Bonferroni correction for adjusting the significance level to see which of the conditions differed significantly from each other. Only response latencies to control words in the low constraint condition differed from all other conditions. There was no difference between cognate latencies in low versus high constraint sentences. Means, standard deviations and confidence intervals are presented in Table 2.

**TABLE 2:** Means and standard deviations of first stroke latencies (in ms) as a function of sentence constraint and cognate status.

	Low Constraint		High Constraint	
	*Mean*	*SD*	*Mean*	*SD*
Controls	1537	234	1263	144
Cognates	1327	147	1363	210

## Discussion

A large body of research suggests that bilingual lexical access is language non-selective (Dijkstra et al., 1999; Costa et al., 2000; Lagrou et al., 2011a). This entails that representations of both languages may be activated simultaneously during word production and recognition, even in a single language context. The cognate facilitation effect has provided evidence for such a hypothesis in written and spoken word recognition as well as in spoken word production. The aim of the current study was to ascertain whether cognate effects also occur in written word production, which has not been previously investigated. It therefore contained pictures of cognate and non-cognate target words embedded within either low or high constraint sentences, which Dutch-English bilinguals had to write in their L2 through typing. First key stroke latencies demonstrated a clear cognate effect for pictures presented in a low constraint condition. That is, cognate words were produced faster than control words, with a mean difference of 210 ms (about 14% faster). However, the cognate effect disappeared in the high constraint sentences, due to the fact that control images were responded to much faster in this condition as compared to the low constraint condition. Response times to cognate images remained the same.

Our results are in line with studies on word recognition (e.g., Schwartz and Kroll, 2006; Van Hell and De Groot, 2008; Libben and Titone, 2009), which reported a cognate facilitation effect for lexical decision in low constraint sentences, but no such effect for high constraint sentences. They are also partially in line with the more similar word production study conducted by Starreveld et al. (2014), as we replicated their findings in a low constraint context, but found no cognate effect in a high constraint context. A possible explanation is that the high constraint sentences in the study by Starreveld et al. were less predictive than those in the current study. Important to note is that Starreveld et al. also found a reduced L2 cognate effect in high constraint context, and no effect whatsoever for L1.

Overall, the results of our study suggest that spoken and written word production are not all that distinct in terms of lexical access. Furthermore, even though response times are longer in general for written production than for spoken production, this may be an artifact of technical specificities, such as visual inspection of the writing. Indeed, a study by Perret and Laganaro (2013) showed that reaction time differences between handwriting and speaking only occur when participants can see and monitor their handwritten production. When they were unable to visually inspect what they were writing, responses took no longer than oral production. Similar conclusion may be drawn for typing, where participants may monitor the position of their fingers on the keys before starting to type.

If we add the current study’s outcome to the body of literature on bilingual lexical activation, especially within a constraining sentence context, we may consider two possible explanations for our findings. First of all, it may be the case that sentence context serves as a language cue for appropriate language selection and heightened activation for representations in that language, thereby reducing the cognate effect. Secondly, and more fittingly for the current and previous findings, pre-activation of the target may take place within a highly predictive sentence context before the target stimulus is shown, reducing and even diminishing the cognate effect in high constraint conditions. In other words, pre-activation of the target may be comparable to the activation caused by the presentation itself. This explanation also accounts for the fact that response times on control words are reduced in high constraint conditions, rather than response times on cognates being augmented. Crucially, this explanation fits perfectly within a theory of bilingual access which is language non-selective.

## Data Availability Statement

The raw data supporting the conclusions of this article will be made available by the authors, without undue reservation.

## Ethics Statement

Ethical review and approval was not required for the study on human participants in accordance with the local legislation and institutional requirements. The patients/participants provided their written informed consent to participate in this study.

## Author Contributions

EW and RC conceptualized the study, with valuable input provided by WD. RC programmed the experiment and collected the data. RC analyzed the results together with EW. EW drafted the manuscript. WD provided critical revisions. All authors contributed to the article and approved the submitted version.

## Conflict of Interest

The authors declare that the research was conducted in the absence of any commercial or financial relationships that could be construed as a potential conflict of interest.
