# Supplementary material for: Hands Down: Cognate Effects Persist During Written Word Production
Source: Front Psychol. 2021 Jul 5;12:647362. doi: 10.3389/fpsyg.2021.647362 (PMC8287723; doi:10.3389/fpsyg.2021.647362)
Supplement: Supplementary file 1 [file Table_1.DOCX]

APPENDIX A

List of all the Words used in the Experiment and their Word Frequencies.

| CW | SUBTLwf | MCW | SUBTLwf | FW | SUBTLwf | PW | SUBTLwf |
| --- | --- | --- | --- | --- | --- | --- | --- |
| baby | 509.37 | boy | 529.82 | barrel | 10.63 | stethoscope | 0.94 |
| balloon | 8.67 | bicycle | 6.61 | bird | 45.45 | hamburger | 7.78 |
| banana | 10.73 | bench | 10.08 | bottle | 50.75 | handcuffs | 5.16 |
| bear | 22.99 | belt | 24.35 | cake | 45.06 | doll | 24.76 |
| bomb | 22.99 | bowl | 21.45 | city | 169.1 | radio | 77.18 |
| bus | 74.18 | bag | 94.04 | chimney | 4.18 | lighthouse | 3.08 |
| cactus | 2.9 | carrot | 3.82 | cow | 25.51 | frog | 11.82 |
| hammer | 12.47 | helmet | 9.47 | crown | 13.69 | apple | 23.67 |
| ladder | 9.25 | lemon | 12.2 | egg | 26.04 | chair | 49.24 |
| lamp | 12.88 | lion | 15.35 | fire | 215.49 | dragon | 19.29 |
| microscope | 2.53 | mushroom | 2.14 | glasses | 33.12 | curtains | 6.29 |
| nest | 11.1 | needle | 11.92 | horse | 92.88 | box | 89.75 |
| paperclip | 0.04 | paintbrush | 0.53 | key | 86.86 | star | 60.99 |
| piano | 24.86 | plate | 25.65 | knife | 46.8 | ear | 32 |
| pirate | 7.35 | pencil | 9.86 | lightning | 14.14 | cross | 55.04 |
| popcorn | 9.12 | potato | 11.29 | mountain | 35.39 |  |  |
| puzzle | 7.33 | pumpkin | 10.84 | rainbow | 7.98 |  |  |
| rose | 53.02 | rain | 48.9 | roof | 35.65 |  |  |
| skateboard | 1.67 | screwdriver | 2.51 | shower | 41.12 |  |  |
| spaghetti | 5.92 | scissors | 6.69 | spoon | 7.61 |  |  |
| tent | 17.49 | towel | 14.16 | tree | 65 |  |  |
| tiger | 18.53 | turtle | 17.04 | unicorn | 2.47 |  |  |
| tractor | 3.73 | toaster | 3.88 | volcano | 3.33 |  |  |
| train | 81 | truck | 72.86 | wall | 70.69 |  |  |
| zebra | 2.51 | zipper | 2.82 | wizard | 10.63 |  |  |

*Note: CW = Cognate Words, MCW = Matched Control Words, FW = Filler Words, PW = Practice Words, SUBTLwf = SUBTLEX frequency per million words*

APPENDIX B

List of all the Sentences used in the Experiment

Practice Sentences (Predictive and Non-Predictive with Cognates and Controls)

| 1 | The children were playing with a fake **stethoscope** so they could pretend to be doctor and patient |
| --- | --- |
| 2 | Fred ordered a McDonald's **hamburger** and some large fries |
| 3 | The police officer realized he forgot his **handcuffs** at home when he got to the police station |
| 4 | The little girl was playing with a **doll** in her room |
| 5 | He turned on the **radio** to listen to the news report about the crash |
| 6 | On the shore of the coast there's a **lighthouse** to guide the sailors safely into the harbor. |
| 7 | In the pond she saw a **frog** jumping straight into the water |
| 8 | Justin could see a worm in the rotten **apple** after he took a bite out of it |
| 9 | As a true gentleman he pulled out a **chair** for his girlfriend at the restaurant |
| 10 | He fought his way past the **dragon** so he could save the princess in the tower |
| 11 | I had to close the **curtains** because the sun was shining on the television screen |
| 12 | He was carrying a cardboard **box** filled with his DVD collection since he was moving |
| 13 | When it gets dark and you see the first **star** of the night, you have to make a wish |
| 14 | On his deathbed, my grandfather whispered something into my **ear** and he told me not to tell anyone else |
| 15 | His grandmother is a strict catholic, she even wears a cross around her neck which she kisses sometimes |

Non-Predictive Sentences with Cognate Words

| 1 | After Nancy took care of the **baby**, she watched some television |
| --- | --- |
| 2 | The young child looked up and saw a **balloon** floating away in the sky |
| 3 | As part of her diet, Miranda eats a **banana** every morning |
| 4 | When you see a **bear,** you have to pretend to be dead |
| 5 | The investigator didn't know where the **bomb** was but luckily it didn't go off |
| 6 | Denis doesn't like taking the **bus** to school but he has no other options |
| 7 | His mother bought him a small **cactus** for his new apartment as a house warming present |
| 8 | He borrowed his father's **hammer** to build a doghouse |
| 9 | The painter stored his **ladder** next to the shed in the backyard |
| 10 | During the fight, a **lamp** got knocked over and shattered on the floor |
| 11 | Sandra bought her son a toy **microscope** for Christmas and her son loved it |
| 12 | The infant pointed enthusiastically at the **nest**, watching the birds fly towards it |
| 13 | The lawyer was playing around with a **paperclip** because he was very nervous |
| 14 | Jeff is very proud of his **piano** because his grandfather made it for him |
| 15 | Stacey liked dressing up as a **pirate** when she was younger |
| 16 | Some people prefer their **popcorn** salted, others like it covered in butter |
| 17 | The student had bought a **puzzle** with a thousand pieces to challenge himself |
| 18 | When Sarah entered the kitchen, she found a **rose** laying on the table |
| 19 | Bob hangs his jacket up and put his **skateboard** against the wall when he got home |
| 20 | There was at least ten different kinds of **spaghetti** on the menu at the Italian restaurant |
| 21 | They walked past a large **tent** which was big enough for six people to sleep in |
| 22 | There is a documentary where a **tiger** chases down an antelope in the savannah |
| 23 | On the side of the road was a **tractor** with smoke coming out of the engine block |
| 24 | Lucy didn't know which **train** to take so she accidentally got on the wrong one |
| 25 | The dentist spotted a **zebra** on his safari trip to Africa |

Predictive Sentences with Cognate Words

| 1 | In the cradle was a crying **baby** who woke up the whole house |
| --- | --- |
| 2 | The clown made an animal out of an inflated **balloon** for the birthday boy |
| 3 | The monkey was peeling the **banana** before he ate it |
| 4 | Winnie the Pooh is a friendly **bear** who loves to eat honey |
| 5 | The terrorist had planted a **bomb** but the police discovered it in time |
| 6 | During a train strike, commuters have to take the **bus** instead of the train |
| 7 | The only plant that grows in the desert is a **cactus** since it doesn't need much water to grow |
| 8 | The woodworker grabbed a nail and used his **hammer** so the pieces of wood would stick together |
| 9 | On top of a fire truck is a giant extendable **ladder** so the fire fighters can get to high places |
| 10 | The room was lit by a standing **lamp** in the corner of the room |
| 11 | Biologists study bacteria using a **microscope** because they are so small |
| 12 | Birds collect small branches and twigs to make a **nest** so they can keep their eggs safe |
| 13 | Some people use a stapler, I prefer using a **paperclip** to keep my documents together |
| 14 | The white keys are larger than the black keys on a **piano** because they are used more often |
| 15 | Captain Jack Sparrow is a infamous **pirate** who sailed on the Black Pearl |
| 16 | At the cinema you always see a lot of people eating **popcorn** even though it is unhealthy |
| 17 | The detective wondered what the missing piece of the **puzzle** was as he looked at his case again |
| 18 | On Valentines day he gave his girlfriend a single red **rose** as a token of his affection. |
| 19 | Tony Hawk is famous for doing tricks with his **skateboard** on halfpipes |
| 20 | Neil loved eating Italian food, like pizza and **spaghetti** but only once per week |
| 21 | If you go camping, you have to put up a **tent** to protect yourself from the environment |
| 22 | Panthers and leopards are cool but the striped **tiger** has to be my favourite large cat. |
| 23 | The farmer was driving his big **tractor** up and down the field to harvest his crops |
| 24 | The conductor checked the tickets of the passengers on the **train** and he fines them if they don't have one. |
| 25 | Is it black with white stripes or is a **zebra** white with black stripes? |

Non-Predictive Sentences with Control Words

| 1 | It wasn't possible for the **boy** to swim across the river. |
| --- | --- |
| 2 | The student tried to find his **bicycle,** but he forgot where he parked it. |
| 3 | He woke up on a **bench** in the park after a night of drinking. |
| 4 | Bill was looking around the room for his **belt** but he couldn't find it . |
| 5 | A pub without beer is like a **bowl** of soup without salt. |
| 6 | The celebrity decided to wear a **bag** on his head to make a statement. |
| 7 | Steven decided to make a **carrot** cake for his mother because it's her favourite cake. |
| 8 | He must have misplaced his **helmet** because he has been searching for it for over half an hour. |
| 9 | The recipe required two scoops of sugar, a **lemon,** two eggs and a lot of love. |
| 10 | His child really wanted to stop at the **lion** enclosure in the zoo. |
| 11 | There is a specific kind of **mushroom** which is absolutely delicious. |
| 12 | You have to throw away the **needle** after using it once, otherwise you might spread diseases. |
| 13 | She picked up the **paintbrush** and made small strokes on the canvas. |
| 14 | The baron ordered his servant to bring him a **plate** so he could throw it at the wall. |
| 15 | The principal opened a drawer to get a **pencil** so he could write a memo. |
| 16 | The rich executive picked up a dirty **potato** with disgust and told his chef to clean it before using it |
| 17 | The farmer presented his prize **pumpkin** at the local vegetable contest. |
| 18 | Vanessa sighed when she saw the **rain** pouring down outside since she forgot her coat. |
| 19 | The thief used a **screwdriver** to break into the car. |
| 20 | You could have used the **scissors** instead of tearing the fabric to pieces. |
| 21 | Tom was wearing a **towel** around his waist when he came out of the bathroom. |
| 22 | Emma was very upset that her **turtle** managed to run away from the garden. |
| 23 | They got a fancy new **toaster** as a wedding present from one of their friends. |
| 24 | The professor got stuck behind a large **truck** in traffic so his university class was going to start late. |
| 25 | The girl told him that his **zipper** was open so he quickly zipped it up. |

Predictive Sentences with Control Words

| 1 | The old man thought back to his childhood, when he was a young **boy** playing football on the street. |
| --- | --- |
| 2 | The Tour de France winner did a victory lap on his **bicycle** after crossing the finishing line. |
| 3 | There were only two people in the park sitting on a **bench** underneath a large tree |
| 4 | His pants kept falling down so he bought a **belt** the very next day. |
| 5 | Mary poured some cereal into a **bowl** before adding the milk. |
| 6 | Evelyn put all her groceries in a brown paper **bag** before paying the cashier. |
| 7 | Bugs Bunny likes eating a **carrot** when he asks Elmer Fudd how he is doing. |
| 8 | To protect your head when cycling, you can wear a **helmet** so you won't get hurt if you fall |
| 9 | Micheal loves drinking cola with a slice of **lemon** because he likes the mix of sweet and sour. |
| 10 | In the jungle, the mighty jungle, the **lion** sleeps tonight. |
| 11 | Kabouter Plop lives in a spotted red **mushroom** in the middle of the forest. |
| 12 | The nurse took a blood sample using a **needle** to check if Peter had a vitamin deficiency. |
| 13 | Picasso used his skill with the **paintbrush** to make wonderful masterpieces. |
| 14 | His mom cooked dinner and put some potatoes and a pork chop on his **plate** before sitting down herself. |
| 15 | The sketch artist paused his drawing to sharpen his **pencil** because the point had broken. |
| 16 | Fried, mashed, cooked, boiled or baked, the **potato** really is a versatile food. |
| 17 | During Halloween, people make scary lanterns out of a **pumpkin** to decorate their houses with. |
| 18 | I always carry an umbrella with me in case of **rain** because I don't want to get my hair wet. |
| 19 | The handy man uses a flathead **screwdriver** to put together the desk. |
| 20 | The hairdresser uses a pair of **scissors** to give me a different hairstyle. |
| 21 | Rachel turned off the shower and dried herself with a fluffy **towel** before using the hairdryer. |
| 22 | It might be a very slow animal, but the hard shell of the **turtle** gives it some form of protection. |
| 23 | For breakfast, Sarah put two slices of bread in the **toaster** and she made some orange juice. |
| 24 | The 18 wheels of the delivery **truck** came to an abrupt halt when the driver braked suddenly. |
| 25 | He tried to close his backpack by pulling on the **zipper** up but it got stuck. |

Non-Predictive Sentences with Filler Words

| 1 | The interior designer used a **barrel** as an improvised table |
| --- | --- |
| 2 | There was a small **bird** sitting outside the window |
| 3 | Lucas forgot he was supposed to bring a **bottle** of wine to the dinner party |
| 4 | His girlfriend quickly hid the **cake** she bought for his birthday before he saw it |
| 5 | Sam had to call somebody to clean the **chimney** because something got stuck inside it |
| 6 | Her boyfriend showed her the **city** when she came over to visit him |
| 7 | Jack made a deal to trade his **cow** for three magic beans |
| 8 | The slave grabbed the **crown** and threw it into the river |
| 9 | The college student grabbed an **egg** and threw it at the politician |
| 10 | The journalist reported about the **fire** that was still ravaging the forests |
| 11 | She had lost her **glasses** when she went rafting on the wild river |
| 12 | There was a scandal about **horse** meat being used in a brand of lasagne |
| 13 | He cursed loudly when he couldn't find his **key** to open the front door |
| 14 | The soldier grabbed the **knife** by the blade and threw it at the enemy |
| 15 | A car drove by right when **lightning** struck the giant oak tree |
| 16 | The surgeon had expressed an interest in **mountain** climbing but he had zero experience in climbing |
| 17 | There was a loud noise coming from the **roof** during the hailstorm |
| 18 | Lisa thought about giving her **saxophone** to a famous jazz musician as a gift |
| 19 | The athlete stepped into the **shower** and washed himself thoroughly |
| 20 | The baby picked up the **spoon** but immediately dropped it on the floor |
| 21 | The archer shot an arrow at the **tree** to practice his aim |
| 22 | The princess rubbed her eyes in disbelief when she saw a **unicorn** running towards her |
| 23 | Josh walked up the **volcano** to throw the ring into the fire so it could be destroyed |
| 24 | She was looking at the **wall** to see if there was any water damage |
| 25 | She didn't believe he was a **wizard,** but he was definitely acting funny |

Predictive Sentences with Filler Words

| 1 | They put the freshly made wine into a large oaken **barrel** to age so the taste would improve |
| --- | --- |
| 2 | I'm not sure whether a parrot or a pelican is my favourite **bird** but I definitely hate pigeons |
| 3 | The whiskey label on the glass **bottle** said the whiskey was aged for over a hundred years |
| 4 | The baker decorated the chocolate birthday **cake** with frosting and candles |
| 5 | Santa Claus got on the roof and climbed down the **chimney** so he could bring gifts to the children |
| 6 | New York is a huge **city** and filled with modern architecture |
| 7 | The farmer went out to milk his last **cow** after all the other ones had run away |
| 8 | A cowboy wears a cowboy hat, a king wears a golden **crown** and a builder wears a hard hat |
| 9 | The chicken with brown feathers lays a brown **egg** every day and never a white egg |
| 10 | The arsonist used a lighter to start a **fire** in the abandoned warehouse |
| 11 | Robin was nearsighted so without his **glasses** he could not read the blackboard |
| 12 | The brave knight was riding his **horse** straight into battle |
| 13 | The building manager unlocked the door using the master **key** since it worked on all the doors in the building |
| 14 | The food critic was eating his steak with a fork and **knife** in the corner of the restaurant |
| 15 | During a thunderstorm you will first see the **lightning** and then hear the thunder |
| 16 | The hiker wanted to climb Everest, the biggest **mountain** on the planet |
| 17 | The technicians were installing solar panels on the **roof** because the owner wanted green energy |
| 18 | The Belgian musician Adolphe Sax invented the **saxophone** which is often used in jazz music |
| 19 | It was a hot day so Tom went into the bathroom to take a cold **shower** so he could cool down |
| 20 | The guest ate her soup with a **spoon** because eating soup with a fork is dumb |
| 21 | In the backyard there was a large apple **tree** which had been there for many years |
| 22 | A horse with an icecream cone on its forehead looks like a **unicorn** from the right angle |
| 23 | Molten lava erupted from the **volcano** and caused massive devastation to the island |
| 24 | The decorator hang a beautiful painting on the brick **wall** on the other side of the room |
| 25 | Harry Potter is a famous **wizard** and he studies at Hogwarts |
